# Supplementary material for: The live attenuated DGAT1-knockout whole-cell Toxoplasma vaccine confers protective immunity against acute and chronic toxoplasmosis
Source: Res Sq. 2026 Aug 27:rs.3.rs-10755545. Preprint. [Version 1] doi: 10.21203/rs.3.rs-10755545/v1 (PMC13533002; doi:10.21203/rs.3.rs-10755545/v1)
Supplement: Supplementary file 1 [file NIHPPRS10755545v1-supplement-1.pdf]

Supplementary Fig. 1. Creation of the *T. gondii* RH ΔDGAT1strain.

(a) Diagram illustrating the strategy to disrupt TgDGAT1 (TGGT1_232730) in the type I *T. gondii* strain using CRISPR/Cas9 gene editing. The location of the guide RNA sequence is shown, indicating the predicted Cas9-induced DNA break in the first exon of TgDGAT1. The repair construct was designed to replace the TgDGAT1 gene with the HXGPRT selection cassette. (b) Diagram demonstrating the PCR screening method to identify *T. gondii* clones with the DGAT1 deletion. One primer pair (P1/P2) is located within the deletion, while another pair (P3/P4) is outside it. The third pair (P5/P6) detects correct integration of the repair template. (c) Representative PCR results from transfected *T. gondii* parasites confirming the deletion of the DGAT1 gene at the genomic level and replacement with the HXGPRT marker. DNA from both parental (WT) and transfected clones was used as a template. Unlike the WT parasites, the ΔDGAT1 clone lacks the P1/P2 band, shows a smaller P3/P4 band, and has a P5/P6 band, indicating successful deletion and correct marker integration. L indicates an 1-kb plus DNA ladder.

Supplementary Fig. 2. *Toxoplasma* RH ΔDGAT1 strain shows improved growth in low-lipid and high-glucose conditions.

The RH WT or RH ΔDGAT1 *T. gondii* strain was cultured in either standard MEM with 10% FBS and 1 g/L glucose or in modified MEM with 10% LPD-FBS and 4.5 g/L glucose. (a) Parasite replication in human fibroblasts was quantified by [^{3}H]-uracil incorporation at 24 hours post-infection, with comparisons between standard and modified media. Results show a slight recovery in replication defects in the absence of lipids and in the presence of added glucose. HFF-1 monolayers grown in both media were infected with either the WT or ΔDGAT1 strain for 24 hours, followed by incubation with tritiated uracil. Data expressed as mean ± SD from 2 independent assays were analyzed using an unpaired two-tailed t-test. (b) Analysis of parasite growth was conducted using plaque assays on fibroblast monolayers after 14 days of infection with 200 RH ΔDGAT1 parasites to compare growth in standard and modified MEM. Mutant parasites showed significantly better growth in the modified medium. Representative images and quantification of the total lysed area and number of plaques (means ± SD) from 3 independent experiments are shown; ****, $p < 0.0001$ (unpaired two-tailed t-test). All scale bars, 5.25 mm.

Supplementary Fig. 3. Inoculation of the ΔDGAT1 mutant induces strong antibody responses against *T. gondii* in mice.

(a) Timeline for detection of *T. gondii*-specific IgG response in BALB/c mice by ELISA. Mice (n = 5 per group) were inoculated with two doses of 10^4 or 10^5 RH ΔDGAT1 parasites, and IgG, IgG1, and IgG2a levels were measured in the serum of naïve and vaccinated mice at 30, 90, and 180 days post-inoculation. Relative levels of *Toxoplasma*-specific IgG were determined by indirect ELISA using immuno-plates coated with 10 µg/mL of soluble *T. gondii* antigens. (b-j) Data for serum dilutions ranging from 1/900 to 1/72900 are shown. Mice immunized with RH ΔDGAT1 showed significantly higher levels of IgG (b, 30 days; e, 90 days; h, 180 days) and its subclasses IgG1 (c, 30 days; f, 90 days; i, 180 days) and IgG2a (d, 30 days; g, 90 days; j, 180 days) than the control group. Statistical analyses were performed using two-way ANOVA with Dunnett's multiple-comparison test. Mean ± SD data with *p*-values are shown.

Supplementary Fig. 4. Vaccination with RH ΔDGAT1 parasites protects mice against acute type I *T. gondii* infection.

(a) Female CD-1 mice (n = 5 per group) received two immunizations of 10^4 RH ΔDGAT1 parasites, two weeks apart, followed by challenge with 500 tachyzoites of the RH luciferase *T. gondii* strain 90 days post-final immunization. (b) Normalized weights (means ± SEM) and (c) survival of naïve and immunized mice were plotted. Vaccinated mice showed no signs of infection or weight loss over 30 days, while naïve mice lost weight rapidly and died quickly by day 11. The log-rank (Mantel–Cox) test assessed survival differences. (d) Bioluminescence images of naïve and immunized mice at days 5, 9, 15, and 21 post-infection. Vaccinated mice exhibited no luminescence at day 21. (e) Whole-body luminescence quantification (mean ± SEM, photons/sec), with *p*-values from multiple unpaired t-tests with Holm-Sidak's

correction. (f) Female CD-1 mice (n = 5 per group) were immunized twice with either 10^3 or 10^4 RH ΔDGAT1 parasites. Thirty days after the last immunization, they were challenged with 500 tachyzoites of the type I RH luciferase-expressing *T. gondii* strain. All vaccinated mice survived the initial challenge and were later rechallenged 90 days afterward. (g) Normalized weights (means ± SEM) and (h) survival for naïve and immunized mice were plotted. Vaccinated mice displayed no infection symptoms or weight loss during 30 days, while naïve mice quickly lost weight and died within 12 days of infection. The log-rank (Mantel–Cox) test was used to assess the statistical significance of the difference in survival probability between naïve and vaccinated mice. (i) Bioluminescence images of naïve and immunized mice are shown at days 6 and 9 post-infection with luciferase-expressing RH parasites. Vaccinated mice exhibited no detectable parasite luminescence. (j) Quantification of the total body luminescence from luciferase-expressing parasites in CD-1 mice, shown in (i). Mean ± SEM data (total flux, photons/sec) were plotted, and *p*-values were determined using multiple unpaired t-tests with Holm-Sidak's correction for multiple comparisons.

Supplementary Fig. 5. Immunization of mice with RH ΔDGAT1 parasites prevents cyst formation due to type II *T. gondii* infection.

(a) Female BALB/c mice (n = 5 per group) were given two immunization doses of 10^3 or 10^4 RH ΔDGAT1 parasites, spaced two weeks apart, followed by challenge with 2000 tachyzoites of the type II Me49 luciferase-expressing *T. gondii* strain 30 days after the final immunization. (b) Normalized weights (means ± SEM) for naïve and immunized mice were plotted for all surviving mice at the specified time. (c) Brain cyst numbers of surviving or moribund mice, euthanized at 30 days or after 2 weeks of infection, respectively, were determined using DBA staining. Mean ± SEM data are shown (*p*-values calculated by one-way ANOVA with Dunnett's multiple-comparison test). (d) Bioluminescence images of naïve and immunized mice are shown on days 4, 11, 18, and 24 after infection with luciferase-expressing Me49 parasites. To assess brain cyst load in chronic infection, mice were imaged dorsally on days 18 and 24. (e) Quantification of whole-body luminescence of Me49-Luc parasites in BALB/c mice, shown in (d). Mean ± SEM data (total flux, photon/sec) were plotted, and *p*-values were obtained using two-way ANOVA with Dunnett's multiple-comparison test.

Supplementary Fig. 6. The immune response in mice following ΔDGAT1 mutant vaccination involves $CD8^+$ T cells.

(a) Female BALB/c mice received two doses of 10^4 RH ΔDGAT1 parasites, two weeks apart. IFN-γ levels, $CD4^+$ T cells, or $CD8^+$ T cells were then depleted via intraperitoneal injections of 200 μg of anti-IFN-γ (Clone XMG1.2), anti-CD4 (Clone GK 1.5), and anti-CD8b (Clone H35-17.2) monoclonal antibodies, or mock isotype controls (Clones HRPN IgG1 and LTF2 IgG2b) on specified days, before and after challenge with a lethal dose of 500 luciferase-expressing type I RH tachyzoites. T-cell responses were measured in the serum of naïve and vaccinated mice on day 0 and day 7 of challenge. Percentages of (b) $CD3^+$ $CD4^+$ T cells and (c) $CD3^+$ $CD8^+$ T cells within peripheral lymphocytes of mice (n = 5 per group) were detected with flow cytometry. Results confirmed the successful depletion of $CD4^+$ and $CD8^+$ T cells in antibody-treated mice before and after infection. Data are presented as means ± SD. Statistical significance was determined using multiple unpaired t-tests with Holm-Sidak's multiple-comparison test.
